# Promise, Mimicry, and Surveillance: Responsibly Integrating Artificial Intelligence With Socratic Inquiry in Medical Education

**DOI:** 10.2196/102958

**Published:** 2026-09-10

**Authors:** José Daniel Sánchez

**Affiliations:** 1 Facultad de Ciencias de la Salud y Bienestar Humano, Universidad Tecnológica Indoamérica, Machala y Sabanilla, Quito, Pichincha Province, 170124, Ecuador, +593 984663224

**Keywords:** AI, medical education, Socratic method, clinical reasoning, large language models, learning analytics, educational ethics

## Abstract

Large language models are increasingly proposed as a way to deliver Socratic dialogue at scale in medical education, offering personalized, always-available, and lower-stakes inquiry that human educators cannot logistically provide. In this viewpoint, I argue that this promise is plausible but unproven and that 2 failure modes deserve more attention than they currently receive. The first is the mimicry trap: a system that fluently generates Socratic-sounding questions can appear to cultivate reasoning while functioning as interactive content delivery. I position this as an educational instance of the ELIZA effect and of the proxy-outcome problem; separating conversational mimicry from genuine metacognitive gain remains a central unresolved evaluation problem. The second is what I term the Panopticon Paradox: the data collection that makes AI tutoring effective may erode the psychological safety that Socratic inquiry requires, pushing learners toward performative rather than authentic engagement, in a manner analogous to gaming behaviors documented in intelligent tutoring systems. I present this second construct as a testable causal model with specified mediators, moderators, and falsifiable predictions rather than as an established finding. Because the evidence base is dominated by proof-of-concept tools, cross-sectional surveys, and short-term evaluations, I argue that AI is complementary rather than a replacement technology, and I propose a 3-pillar framework (governance, curriculum, and faculty development) tied explicitly to the 2 failure modes. I distinguish 4 modalities of delegation and argue that ethical deliberation, emotionally complex communication, and ambiguous clinical judgment cannot presently be recommended for autonomous or primary AI delivery.

## Introduction: The Allure of an Untiring Socrates

Medical education rests on a standing tension: an expanding knowledge base must be transmitted while the critical thinking required for clinical practice is cultivated [1]. The Socratic method has long been the pedagogy of choice for the second task, promoting reasoning through structured, question-driven dialogue that mirrors the analytic uncertainty of clinical work [2,3]. However, authentic Socratic teaching is time-intensive, hard to scale beyond one-on-one interaction, and chronically vulnerable to degrading into “pimping”: rapid-fire, evaluative questioning that humiliates rather than teaches [2].

Against this structural backdrop, large language models (LLMs) are attractive precisely because they appear to relieve the tension between scale and pedagogical quality. They sustain extended dialogue, adapt to a learner’s responses, and do so on demand and in private, at scales human educators cannot achieve [4,5]. Where infrastructure and licensing permit, the marginal cost per additional learner is low relative to faculty time, although acquisition, integration, and oversight costs are not negligible and access is unevenly distributed across health systems [6]. The pitch writes itself: an untiring, patient Socrates for every learner, available at any hour, in multiple languages, with no enrollment limit. Some of this promise has pedagogical substance. Well-designed AI dialogue can reduce evaluation anxiety and provide immediate feedback on reasoning, and learners in several studies report a lower-stakes space in which to reason aloud [7,8]—though whether a given system is experienced as nonjudgmental depends on its design, on how the institution uses its outputs, and on what learners believe happens to their data.

My aim is not to dismiss the promise or to call for abandoning AI in medical education. It is to argue that deployment is outpacing evidence [6,9] and that 2 specific failure modes—the mimicry trap and the Panopticon Paradox—are underexamined precisely because the promise is attractive and the resource pressure is acute. Naming these problems and situating them within existing scholarly traditions is a precondition for responsible integration [10]. This argument is directed at medical educators, curriculum designers, and institutional leaders who decide how and under what safeguards AI tutoring enters medical curricula.

A word on the evidence base: this is a viewpoint, not a systematic review, and the literature was assembled narratively and selectively. I searched PubMed, Scopus, and Google Scholar using terms combining AI, LLMs, and generative AI with medical education, the Socratic method, clinical reasoning, learning analytics, and educational technology governance; the last search was performed in December 2025, with targeted updates through March 2026. I prioritized records published from 2019 onward, supplemented by older sources from which the underlying constructs originated. Selection was purposive, so the evidence cited should be read as illustrative rather than exhaustive; systematic syntheses of this literature are available elsewhere [6,9].

## What Authentic Socratic Teaching Requires

Before asking whether AI can emulate Socratic teaching, it is worth being precise about the target because much of the optimism in the field rests on emulating the wrong one. Genuine Socratic dialogue is constructivist: a shared inquiry in which the instructor is a facilitator rather than an information source, using sequenced, probing questions to surface and examine a learner’s underlying beliefs and reasoning processes [2,3]. It is not question-asking; it is a deliberate strategy to shift intellectual agency from teacher to learner. It targets analysis, evaluation, and synthesis, and it functions as a metacognitive tool, helping learners recognize the limits of their knowledge and develop self-directed inquiry habits [3]. The psychological contract is explicit: the teacher creates a space in which uncertainty is expected and productive, and questions are invitations to think rather than traps to expose ignorance [2,11].

Because the current evidence bears unevenly on the outcomes claimed for AI tutoring, 4 constructs must be kept distinct throughout. Clinical reasoning is the situated capacity to generate, prioritize, and revise diagnostic and management hypotheses in authentic cases under uncertainty; it is typically assessed through script concordance, key-features testing, or workplace-based observation. Critical thinking is the more general higher-order analysis and evaluation of claims and evidence, usually assessed with domain-general instruments. Metacognition is the capacity to monitor and regulate one’s own thinking, including the calibration of confidence to accuracy. Self-directed learning is the autonomous identification of learning needs and the pursuit of resources to meet them. These constructs are correlated but not interchangeable, and the distinction is consequential: a trial that improves self-directed learning scores has not thereby demonstrated an effect on clinical reasoning [7].

The corrupted twin of Socratic teaching, “pimping,” superficially resembles it but inverts nearly every element. Where Socratic questions are process-oriented, open-ended, and lead toward synthesis, pimping questions are closed, often deliberately obscure, asked before an audience, and serve to expose ignorance and assert hierarchy [2]. The distinction is psychological rather than stylistic: authentic dialogue creates productive discomfort inside a psychologically safe space, whereas pimping creates fear, shame, and defensive withdrawal. Psychological safety here is not a loose metaphor but a measurable construct, the shared belief that the setting is safe for interpersonal risk-taking such as admitting error, asking for help, or raising a doubt, for which validated scales exist [11]. The prevalence of pimping is not merely a failure of individual character; it is a rational adaptation to structural constraints, namely large cohorts, chronic time pressure, and the simultaneous mandate to teach and to evaluate summatively [2,12]. Given these pressures, the realistic alternative to AI tutoring is often not ideal one-to-one human tutoring but a lecture hall or a pimping session, and that comparison is the strongest argument for AI in medical education [4,5]. Whether AI fills the gap pedagogically is the question to which I now turn.

## The Mimicry Trap: Fluency Is Not Pedagogy

A consequential risk in the current literature is to treat the generation of Socratic-sounding questions as equivalent to Socratic teaching; several widely discussed systems are characterized by the dialogue they can produce rather than by any measured effect on reasoning [13,14]. An LLM can produce an adaptive, grammatically sophisticated, and well-ordered sequence of follow-up questions without any of this contributing to the learner’s metacognition or reasoning development; a dialogue can be fluent, contextually responsive, and pedagogically inert at the same time [15]. I call this failure mode the mimicry trap: the risk that a system fluent enough to sound Socratic will be mistaken for Socratic teaching, and that measures of dialogue quality—comprehensibility, coherence, adaptivity, and learner satisfaction—will be mistaken for measures of learning.

The construct is not novel, and I do not claim it as such. It is an educational instance of what has been called the ELIZA effect: the tendency to attribute understanding and intent to a system that merely produces human-like output. Weizenbaum’s original demonstration showed that users attributed comprehension and even therapeutic concern to a pattern-matching script with no model of meaning whatsoever [16], an attribution later named and analyzed as a general property of human interaction with symbolic systems [17]. It is also a variant of the proxy-outcome problem: when performance on an easily quantified indicator is substituted for the construct of interest, the indicator degrades as a measure precisely because it has become a target [18,19]. In AI-supported Socratic education, dialogue quality is the proxy and durable reasoning is the construct. Distinguishing genuine, durable gains in metacognition and clinical reasoning from conversational mimicry is, I contend, a central and still largely unresolved evaluation problem in this field [15].

The current evidence does not resolve it. Recent systematic reviews and meta-analyses of AI-supported problem-based and case-based learning report encouraging but heterogeneous effects [20,21], while reviews of AI-enabled tools stress that rigorous, comparative, and longitudinal data remain scarce [9]. A systematic review addressing AI-enabled tools and clinical reasoning in undergraduate nursing education concluded that the available studies are few, heterogeneous in design and outcome measurement, and methodologically limited [22]. The strongest individual signals are real but narrow. A randomized controlled trial of custom GPT facilitation improved self-directed learning scores and assessed critical thinking with a domain-general instrument but did not measure clinical reasoning in authentic cases [7]. A quasi-experimental Socratic AI platform improved self-efficacy but yielded no statistically significant change in critical thinking at a single site over a short interval [8]. ChatGPT-assisted problem-based learning raised theoretical knowledge and mini-clinical evaluation exercise scores in one controlled cohort of urology interns [23]. Named implementations are proliferating—generative teaching assistants [24], Socratic voice assistants [25], customized tutors such as AnatomyGPT and Anatbuddy [26,27], teacher-AI-student models [28], and case companions built explicitly on Socratic dialogue [29]—but these are predominantly pilots, perception surveys, and proof-of-concept systems. Some frequently discussed demonstrations are not peer reviewed: an adaptive Socratic tutor built on clinical pathological conference cases offers an instructive architecture but exists as a preprint without an outcome trial [13], and a blueprint for AI patient simulation using the Socratic method describes the mechanism rather than the measured effect [14]. Cross-sectional studies show high student readiness and a clear training need but cannot establish causation [30,31].

Altogether, these studies justify cautious optimism about engagement, satisfaction, and self-efficacy and considerably less about durable clinical reasoning. Transferable diagnostic reasoning is not entirely unmeasured, but the existing evidence is limited in volume, heterogeneous in how the construct is operationalized, and methodologically weak, with few longitudinal or multisite comparisons [9,22]. Conflating proximal with distal outcomes is how a genuinely promising tool gets oversold, and it is exactly what the mimicry trap predicts.

## The Panopticon Paradox: When Datafication Undermines Inquiry

### The Paradox Stated

Suppose the mimicry problem was solved and AI tutoring did reliably foster reasoning. A second tension would remain. The capability that makes AI tutoring powerful—continuous logging of learner behavior, fine-grained tracking of comprehension, recording of wrong answers and conceptual stumbles, algorithmic analysis of query patterns—is also the mechanism by which it may undermine the psychological safety that Socratic inquiry requires. I call this the Panopticon Paradox, borrowing Foucault’s account of the prison in which inmates, unable to know when they are observed, internalize the possibility of observation, and modify their behavior toward compliance [32]. I offer it as a hypothesis to be tested, not as a validated construct.

The pedagogical promise of AI tutoring is that it dissolves the pimping problem by providing a private space in which learners can fail safely [7,8]. However, the personalization that makes such tutoring effective requires pervasive data collection: a tutor that adapts to evolving misconceptions must store those misconceptions; one that scaffolds must retain a record of what the learner struggled with; one that paces adaptively must log every interaction with timestamps and outcomes [24,33]. These data are genuinely valuable for pedagogy. They are also sensitive because they reveal not only knowledge gaps but also episodic confusion, trial-and-error reasoning, and dead ends that learners may reasonably wish to have forgotten once resolved [33]. If learners come to believe that their practice space feeds institutional models, contributing to grades, permanent profiles, or opaque ranking systems, the incentive structure changes. The deliberate admission of not knowing the essential first move in Socratic dialogue becomes risky [11]. The learner shifts from a participant in inquiry to an actor in a performance.

This prediction is not speculative in its behavioral component. Learners in monitored digital learning environments are well documented to develop strategies that satisfy the system rather than the learning objective, a family of behaviors characterized in the intelligent tutoring literature as gaming the system [34]. Scholarship on learning analytics has similarly argued that continuous predictive modeling reframes students as data points rather than agents, with consequences for vulnerability and consent [33], and qualitative work on AI in higher education documents both dependency and ambivalence among educators asked to adopt these systems [35]. What has not been tested, to my knowledge, is the specific pedagogical claim: that perceived surveillance in an AI tutor degrades reasoning outcomes through a reduction in psychological safety.

### Four Constructs That Are Routinely Conflated: Data Security, Confidentiality, Psychological Privacy, and Freedom from Institutional Assessment

Discussions of “privacy” in educational AI collapse 4 distinct constructs, and the paradox described here depends on the difference between them. Data security is the technical protection of stored and transmitted data against unauthorized access [36]. Confidentiality is the set of rules governing who may legitimately access identifiable data and for what purpose [36,37]. Psychological privacy is the learner’s subjective sense of an unobserved space in which thought can be provisional; it is a perception, and it can be absent even when security and confidentiality are impeccable [11,33]. Freedom from institutional assessment is a structural property of the system: whether the record of a learning interaction can, in principle, be used in a summative judgment [33,38]. The pedagogical mechanism runs through the last 2, not the first 2. A system can be encrypted, compliant, and access-controlled while still being experienced as a place where mistakes count, and it is that experience, not the cryptography, that determines whether a learner admits confusion [11].

### A Testable Model: Causal Sequence

Stated as a causal sequence, the paradox runs: data collection intensity and its visibility to learners increase perceived surveillance [32,33]; perceived surveillance reduces psychological safety [11]; reduced psychological safety reduces authentic inquiry behaviors—help-seeking, admission of uncertainty, exploratory and speculative questioning, tolerance of being wrong in the record [11,34]; and reduced authentic inquiry reduces reasoning transfer to novel cases [3]. Perceived surveillance and psychological safety are the proposed mediators; the model predicts that any effect of tracking on reasoning transfer is fully or partly mediated through them, and that an intervention that reduces perceived surveillance without changing the underlying data flows will still improve outcomes.

Each component is measurable with existing instruments or straightforward adaptations: data collection intensity by system configuration and log volume; perceived surveillance by self-report scales adapted from workplace monitoring research; psychological safety by established team and learning-climate scales [11]; authentic inquiry behaviors by log-derived indicators of help-seeking, hedged or exploratory phrasing, and question initiation, together with gaming-detection approaches developed for intelligent tutoring systems [34]; and reasoning transfer by performance on novel, clinically authentic cases not covered in AI-tutored practice. The proposed moderators are the transparency of the data policy, the presence or absence of an enforced practice-assessment boundary, the retention period, learner stage and the stakes of the surrounding curriculum, and baseline institutional trust.

**Figure 1.**
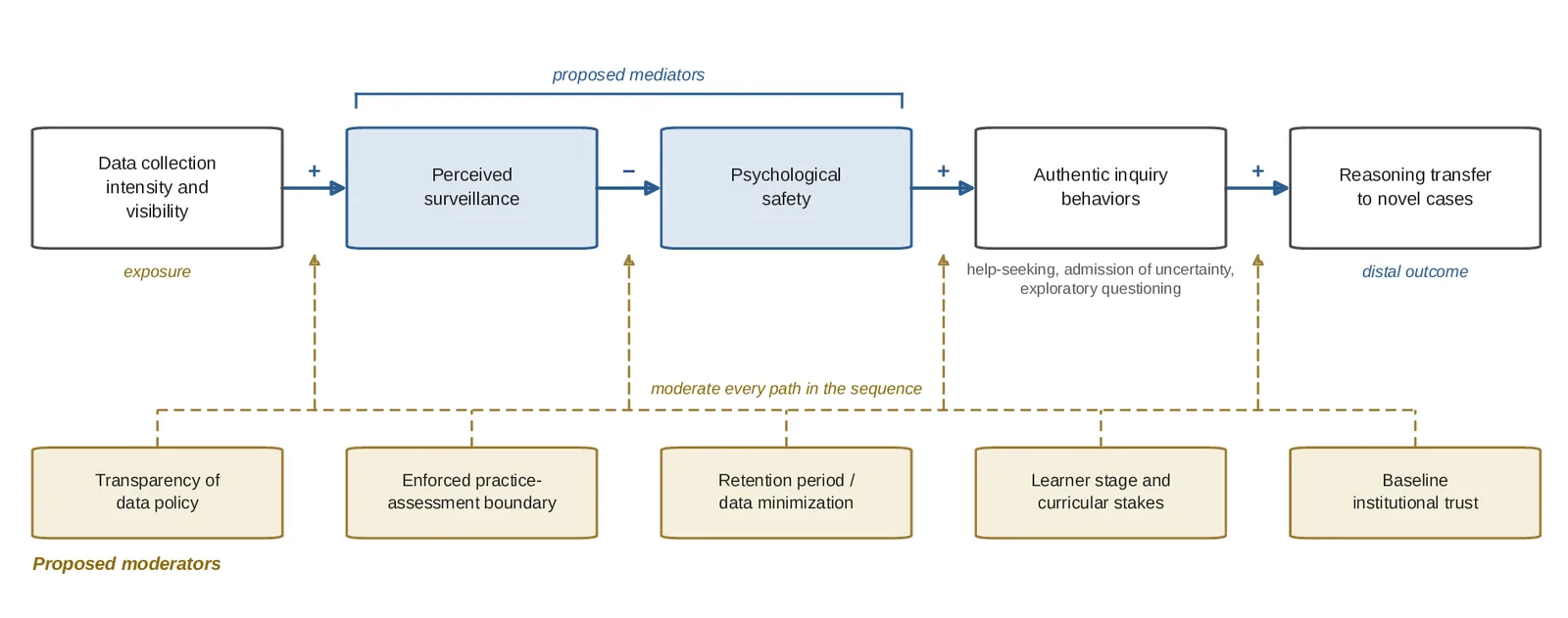
Proposed causal sequence of the Panopticon Paradox, with mediators, moderators, and the direction of predicted effects. The model is advanced as a falsifiable hypothesis; the predictions it generates are stated in the text. Signs indicate the predicted direction of effect. The model predicts full or partial mediation: any effect of tracking on reasoning transfer should operate through perceived surveillance and psychological safety rather than through interaction volume.

The model yields falsifiable predictions. First, learners randomized to identical AI tutors with visible tracking will admit uncertainty less often and produce fewer exploratory questions than learners in an untracked condition, with a corresponding difference in transfer performance [11,34]. Second, this difference will attenuate when a practice-assessment boundary is credibly communicated and enforced [33,38]. Third, mediation analysis will show that the effect of tracking on transfer runs through perceived surveillance and psychological safety rather than through engagement volume. Fourth, the effect will be larger in high-stakes curricular contexts and among learners with lower institutional trust [33]. If a well-powered study finds no difference in uncertainty admission between tracked and untracked conditions, the model as stated is wrong, and the governance recommendations that follow from it lose their empirical warrant. Figure 1 summarizes the proposed sequence, its mediators, and its moderators.

### Why Data Minimization Is a Pedagogical Constraint

None of the available mitigations are complete. Transparent disclosure with opt-in consent, a clean separation between untracked practice and tracked assessment, and governance requiring deletion of individual interaction data each addresses part of the problem while leaving the core tension intact [33,38]. The practical lesson is that data minimization should be justified pedagogically, not only legally. In the United States, the Family Educational Rights and Privacy Act (FERPA) governs education records and grants specific rights over them [37]; the European Union’s General Data Protection Regulation imposes a distinct, and in several respects, stricter regime, including an explicit data minimization principle [36]. These regimes are not equivalent, and an institution outside the United States cannot treat FERPA compliance as a benchmark. The pedagogical rationale, by contrast, is jurisdiction-independent: collecting less data reduces perceived surveillance, which is the mechanism through which the paradox operates. An institution in a jurisdiction with no applicable statute has the same pedagogical reason to minimize collection as one bound by the strictest.

## Where AI Should Not Be the Primary Modality: A Concrete Case

A serious position on integration must specify not only where AI helps but also where it should not be trusted as a primary modality. Based on current evidence, AI-based Socratic tutoring is most defensible for low-stakes conceptual rehearsal: foundational-science questioning, adaptive case prompting, and formative practice in which errors are inexpensive and human review remains available [4,6,39]. It is least defensible in domains that hinge on human judgment and relationship: ethical deliberation, emotionally complex communication such as breaking bad news, ambiguous clinical judgment under uncertainty, and patient-centered nuance [40,41].

Two limitations underwrite this position, and both require careful statements. The first is factual reliability. LLMs are optimized for linguistic plausibility rather than truth and can produce confident, fluent, and incorrect guidance. Direct evaluations in medical domains bear this out with useful specificity: expert raters scored a general-purpose model’s factual accuracy on thoracic anatomy queries significantly below that of a retrieval-augmented tutor constrained to a curated knowledge base [27], and the systematic human evaluation of medical question answering has found that model outputs, even from domain-adapted systems, contain content that raters judged potentially harmful at nontrivial rates [42]. Models have also been shown to reproduce discredited race-based clinical claims, which matter both for accuracy and for equity [43].

The second is empathy, and here, the evidence is less straightforward than the usual claim allows. Blinded raters have judged chatbot responses to patient questions as more empathic than physician responses in a public-forum comparison [44], which should caution against asserting a simple empathy deficit. What that study demonstrates is that text can be rated as empathic; it does not demonstrate that a system holds a model of another person’s difficulty, sustains a longitudinal relationship, or bears responsibility for it. The relevant limitation is therefore not the production of empathic language but the absence of the relational and moral substrate that clinical empathy denotes [40,41]. In domains where that substrate is the object of instruction, AI is a poor primary teacher regardless of how warm its prose is. “AI should complement, not replace, the educator” is a familiar slogan; it becomes meaningful only when made this specific.

A concrete case holds the distinction in view. Consider a learner rehearsing a conversation in which a patient with newly diagnosed metastatic disease asks whether treatment is worth pursuing. An LLM can generate a plausible patient turn, respond to the learner’s phrasing, and offer feedback that reads as thoughtful [44]. What it cannot do is register that the learner’s voice changed, that the pause before the answer was the significant event, or that the learner deflected into prognostic statistics because the emotional content was intolerable; nor can it hold the learner accountable in the way a supervisor who will see them on the ward tomorrow can [40,41]. The same tool used differently is defensible: generating the scenario, providing the learner unlimited low-stakes repetitions, and then handing the transcript to a faculty member for debriefing places the AI where its capabilities lie and keeps the interpretive and relational work with a human [39]. The question is never whether AI touches a domain, but which function within that domain it is asked to perform and who reviews the result.

## A 3-Pillar Framework for Responsible Integration: Governance, Curriculum, and Faculty Development

If the goal is human-AI symbiosis rather than substitution, good intentions are insufficient. I propose 3 interdependent pillars, each with specific controls, accountable actors, and evaluation measures [10,39]. Triadic models of technology adoption are not novel; the contribution here is to tie each pillar to the 2 failure modes above rather than applying generic governance templates to a nongeneric tool.

Governance addresses the Panopticon Paradox. Institutions should move beyond point-of-purchase vendor agreements to sustained multidisciplinary oversight by convening bodies that include educators, technical experts able to explain model behavior, data-governance specialists, legal counsel, and learner representatives [10,33]. Before acquisition, institutions should adopt responsible-use principles such as those articulated by the Association of American Medical Colleges [10] and require the documented assessment of the specific educational gap being addressed; a data inventory specifying what is collected, how long it is retained, with whom it is shared, and when it is deleted [36]; system limitations and the thresholds at which a tool is withdrawn; a cost-benefit analysis that accounts for opportunity cost and the value of displaced educator time; and named actors accountable for ongoing audit. Two structural decisions follow from the model in Figure 1. Data minimization means committing as a policy to collect only what the system needs to function, not comprehensive behavioral data for secondary analytics [36,38]. Practice-assessment separation means enforcing a categorical boundary between formative interaction data and data usable for grades, competency judgments, or certification and communicating that boundary to learners in terms they find credible, since it is perceived surveillance, not the data architecture alone, that the model identifies as the operative mediator [11,33].

The curriculum counters the mimicry trap. AI literacy should be a declared, teachable competency taught from the beginning of training: learners should understand the capabilities and limitations of dialogue systems, their propensity for confident factual error, and the mechanisms by which they can appear more competent than they are [10,42]. Learners should also be taught the ELIZA effect and the proxy-outcome problem explicitly, so that they can evaluate claims about AI tutoring, including their own impressions of it [16,18,19]. Implementation should be phased by stakes and cognitive level: Socratic AI tools are well suited to foundational, lower-stakes contexts in preclinical years and early rotations and should shift to a supplementary role, embedded in human-led teaching and followed by faculty debriefing, as stakes rise [39]. Most importantly, assessment must target what matters rather than what is easy to capture. Programs should assess reasoning transfer, that is, application to novel and clinically authentic cases not covered in AI-tutored practice, together with metacognitive development [3,20]. These are harder to measure than satisfaction, which is precisely why the field defaults to satisfaction [9,21].

Faculty development enables both. Educators need systematic upskilling in the critical appraisal of AI tools, in the verification of AI-generated content, and in a shifted pedagogical role that moves from content delivery toward facilitation and debriefing. This shift is not trivial: it requires faculty to model the intellectual humility and ethical reasoning that AI cannot supply and to bridge between AI-mediated practice and authentic clinical reasoning. Recent guidance on clinical supervision of AI use maps directly onto the educational setting, where the supervisor ensures the tool is used appropriately, interprets outputs correctly, and escalates to human judgment when uncertainty demands it [39]. Research on faculty perceptions consistently finds interest paired with low confidence and limited training [12,45], which makes this pillar the most frequently underfunded and the most likely point of failure.

It is worth distinguishing 4 modalities of AI use, each with a different risk profile [10,39]. Autonomous use, in which AI determines judgments without human oversight, is the highest-risk category and should be avoided. AI as a primary instructional modality, in which learners receive core instruction from AI with human engagement supplementary, is of lower risk but remains problematic for high-stakes reasoning and ethical deliberation [42,43]. Supervised AI-supported practice, in which learners work with AI dialogue under faculty oversight, is substantially of lower risk and aligns with the human-centered model advocated here [39]. Simulated scenarios followed by structured human debriefing are of lowest risk and perhaps of highest value because they preserve the faculty-learner relationship and the interpretive work in which reasoning develops [40,41]. The framework supports the third and fourth modalities, the second cautiously and only in foundational contexts, and the first not at all.

A word is owed to institutions for which this framework may read as written for someone else. The governance apparatus described above presupposes administrative capacity that many schools in low-income and middle-income settings do not have, and the resource pressure that makes AI tutoring attractive is most acute precisely where that capacity is thinnest [6,9]. Two points are as follows. First, the 2 structural decisions that matter most for the Panopticon Paradox, data minimization and practice-assessment separation, are policy choices rather than expensive infrastructure; a school can adopt both in a single faculty-council decision and communicate them to learners in a paragraph [36,38]. Second, dependence on vendors headquartered in other jurisdictions raises questions about where learner data reside and under whose law, which cannot be resolved by pointing to the vendor’s compliance with a foreign statute [36,37]. Institutions in these settings have the strongest interest in minimal collection and the weakest leverage to negotiate it.

## Limitations

This viewpoint has limitations that bear on how its claims should be weighed. The literature it draws on was selected purposively rather than systematically, and I may have favored work consonant with the critical framing advanced here; no protocol, dual screening, or gray-literature search was undertaken, and none of the studies cited were appraised with a formal risk-of-bias instrument [6]. Both central constructs are conceptual proposals rather than validated models: the mimicry trap reframes an existing problem in measurement [18,19], and the Panopticon Paradox, though stated here in testable form, has not been tested. The framework I propose is offered as a reasoned structure for institutional decision-making, not as an intervention with demonstrated effects on learning outcomes.

The relative strengths and weaknesses that justify this complementary division of labor are summarized in Table 1. Because the claims in such comparisons are frequently asserted rather than demonstrated, each row identifies whether the AI-side claim rests on direct empirical evidence or is a reasoned expectation awaiting test.

**Table 1.** Attributes of human-led vs AI-driven Socratic teaching: a context-dependent heuristic, not a meta-analysis or a systematic comparison of effect sizes^a^.

Attribute	Human-led Socratic teaching	AI-driven Socratic dialogue	Basis of the AI-side claim
Scalability	Intrinsically limited to small groups or one-to-one dialogue [2]	Scales to large cohorts; marginal cost per additional learner is low once infrastructure and licensing are in place [5]	Reasoned expectation; cost claims are context-dependent and rarely audited against displaced faculty time
Availability	Time-dependent and schedule-dependent [2]	Continuous and on demand where connectivity, licensing, and devices permit [4]	Empirical as a system property; conditional on access, which is unevenly distributed
Personalization	High in dyadic settings; limited at scale [3]	Adapts to learner responses and prior performance within a session, as described in system reports and narrative overviews [24,29,45]	Reported capability rather than direct evidence; adaptation within a Socratic tutoring system has been described rather than directly evaluated, and its effect on learning outcomes is untested
Consistency	Varies with instructor state, expertise, and bias [12]	Configurable and reproducible across sessions and cohorts [14]	Reasoned expectation; consistency is not neutrality, and models carry biases of their own [43]
Psychological safety	Variable: can be high, or humiliating where it degrades into pimping [2]	Potentially high, contingent on data minimization, an enforced practice-assessment boundary, and what learners believe about data use	Hypothesis, not demonstrated; this is the mechanism that the Panopticon Paradox predicts can fail
Empathy and authenticity	Present; reads emotional and social cues and carries relational responsibility [40]	Can generate text that blinded raters judge as highly empathic, without a relational or moral substrate [44]	Empirical for rated text quality; the limitation concerns relationship and accountability, not prose [40,41]
Content accuracy	Generally high when guided by expert knowledge [6]	Variable and prone to plausible-sounding factual error; accuracy improves when the model is constrained to a curated knowledge base [27,42]	Empirical, from the direct expert rating of model outputs in medical domains
Reasoning transfer	The declared goal of the method; assessed through workplace-based observation [3]	Not established; few longitudinal or comparative studies report transfer outcomes [9,22]	Evidence gap rather than a claim in either direction

a Entries reflect tendencies reported across the cited literature; variation within each modality is considerable and depends on implementation quality, learner characteristics, and institutional context. The last column states whether the AI-side claim rests on evidence directly demonstrated by the cited source or is a reasoned expectation awaiting test.

## A Research Agenda

The priorities follow from the analysis. First, longitudinal, multi-institutional studies that track clinical reasoning over academic years rather than weeks and that determine whether Socratic AI dialogue produces durable gains in diagnostic reasoning and transfer to novel problems are needed [9,22]. Comparative trials should disaggregate the components of AI tutoring by design, separating conversational fluency from reasoning development; without that separation, the field will converge on dialogue quality as a proxy for pedagogical effect [18,19], which is the mimicry trap operating at the level of an entire literature.

Second, equity and algorithmic-bias audits are needed, with attention to generalization across diverse learner populations and low-income and middle-income educational contexts. Most current evidence derives from high-income, English-language, well-resourced schools [6,9]. This is not only an ethical problem but also a scientific one: models trained predominantly on English text and North American norms may perform differently for learners with other linguistic, cultural, and epistemological backgrounds and have been shown to reproduce biased clinical content [43].

Third, and most directly tied to the Panopticon Paradox, the causal model in Figure 1 should be tested. Designs comparing AI-tutored learning in tracked vs untracked conditions, with the measurement of perceived surveillance, psychological safety, help-seeking, uncertainty admission, and reasoning transfer, would either support the mediation pathway or falsify it [11,34]. Governance models—data minimization, practice-assessment separation, and transparency to learners—should be tested as moderators rather than assumed to work [33,38].

Finally, this program should engage philosophers, ethicists, and learners themselves, not only cognitive scientists and educationalists [33,38]. What is the relationship between feeling safe and being safe in a datafied learning environment? What do learners and institutions owe each other in terms of authentic participation and privacy? These are not questions that trials alone can answer.

## Conclusions

The case for AI in Socratic medical education is strong enough to take seriously and weak enough to demand restraint. The honest reading of the current evidence base is that AI shows promise in improving engagement, satisfaction, and self-efficacy [7,8,20,21]; that it plausibly complements educators in overstretched systems [4,5]; and that it has not been convincingly shown to build durable, transferable clinical reasoning that persists over longitudinal follow-up [9,22]. The existing studies are few, heterogeneous, and methodologically limited, and the most cited demonstrations are frequently pilots or preprints rather than outcome trials [13,14]. That is not an argument for abandonment. It is an argument for proportionality between what is claimed and what has been shown.

The 2 failure modes I have emphasized are not exotic risks. The mimicry trap is the ordinary consequence of measuring what is easy to measure: a system that produces fluent, adaptive, Socratic-sounding dialogue will generate excellent proxy metrics whether or not it changes how a learner reasons about a patient, and every incentive in procurement, publication, and program evaluation rewards the proxy [18,19]. The Panopticon Paradox is the ordinary consequence of building personalization on observation: the same logs that let a tutor adapt are also the logs that tell a learner their confusion is on record [32,33]. Neither failure requires bad faith. Both are structural, which is why both must be addressed structurally [10,39].

I have tried to state the second construct in a form that can be shown to be wrong. If learners in tracked and untracked conditions admit uncertainty at the same rate, if psychological safety does not mediate the relationship between tracking and reasoning transfer [11], or if a credible practice-assessment boundary makes no difference to either of these, then the Panopticon Paradox is a compelling metaphor with no empirical content, and the governance recommendations that rest on it should be revised accordingly. I would regard such a finding as a contribution because the alternative, deploying surveillance-intensive tutoring at scale on the assumption that observation is pedagogically neutral, is a much more expensive way to find out.

What follows practically is modest and specific. Institutions considering Socratic AI tutoring should ask what educational gap the tool addresses, what data it collects and for how long, whether formative interaction data are structurally walled off from summative judgment, whether learners believe that wall exists, and how the program will detect the difference between fluent dialogue and improved reasoning [10,39]. Educators should treat AI dialogue as a rehearsal space subordinate to human debriefing, not as a substitute teacher. Researchers should measure transfer, not satisfaction [9,22]. AI in Socratic medical education is a beginning, not an end point, and the discipline it demands is the same discipline the Socratic method itself teaches: a willingness to keep asking what we actually know [2,3].

## Abbreviations

FERPA: Family Educational Rights and Privacy Act
LLM: large language model
